# Interpretation for practice guidelines for prevention, diagnosis, and treatment of ventilator-associated pneumonia in burn patients by american burn association

**DOI:** 10.1186/s41038-015-0009-5

**Published:** 2015-08-01

**Authors:** Jie Luo, Guang-hua Guo

**Affiliations:** Department of Burns, the First Affiliated Hospital of Nanchang University, Nanchang, 330006 Jiangxi China

**Keywords:** Ventilator-associated pneumonia, Inhalation injury, Burn, Burn ICU

## Abstract

“American Burn Association Practice Guidelines for Prevention, Diagnosis, and Treatment of Ventilator-Associated Pneumonia in Burn Patients” was published to provide recommendation for the prevention, diagnosis, and treatment of ventilator-associated pneumonia in burn patients. This article makes interpretations and conclusions for prevention, diagnosis and treatment from this guideline in the combination of domestic burn patients.

## Introduction

Ventilator-Associated Pneumonia (VAP) is an infectious disease of pulmonary parenchym that occurs 48 hours or longer after mechanical ventilation by means of endotracheal tubes or tracheostomy. The purpose of “American Burn Association Practice Guidelines for Prevention, Diagnosis, and Treatment of Ventilator-Associated Pneumonia in Burn Patients” is to review the published literature on ventilator-associated pneumonia (VAP) in burn patients [1]. Evidence-based recommendations are provided for the prevention, diagnosis, and treatment of VAP in adult burn patients.

## Review

### The pathogenesis and predisposing factors of VAP

#### Aspiration from respiratory and gastrointestinal colonized bacteria

VAP is often induced by microbial invasion of the sterile lower respiratory tract and lung parenchyma as patients inhale, and less by bloodstream spread or direct extension of adjacent infection. The research has demonstrated that the oropharynx is a potential reservoir for VAP after comparing bacterial DNA samples from broncheoalveolar lavages to organisms present on patients’ tongues [2, 3]. The stomach and sinuses are potential reservoirs of bacteria as well [4].The stomach often gets colonized with Gram-negative bacteria during critical illness, and enteric Gram-negative bacteria are one of the most frequent microorganisms isolated from cultures of patients with VAP. Indwelling gastric tubes weaken the function of the lower esophageal sphincter during mechanical ventilation, resulting in high incidence of gastric reflux and sustained aspiration which evolve the infection in the lung parenchyma.

#### Aspiration from secretions of upper respiratory tract

Bypassing the glottis barrier, with aspiration of contaminated secretions around the endotracheal tube cuff, intubation and mechanical ventilation weaken the normal respiratory tract defenses against infection. The patients with mechanical ventilation are unable to clear their own secretions, which has impaired mucocilary clearance, and are subjected to repeated infection inspite of suctioning. The incidence of VAP increases with the duration of mechanical ventilation. In the early period of ventilation, the risk of VAP increase fastestly, at an estimated incidence of 3 % per day during the first 5 days, decreasing to 2 % per day on days 5 to 10, and to 1 % per day after 10 days [5].

#### Burn and inhalation injury

Burn injury is a strong predisposing factor for VAP. Nearly 10 to 20 % of burn patients are also sufferers of inhalation injury, and the risk increases with increasing burn size [6]. Inhalation injury is a potential signal of prolonged ventilator dependence, increased duration of hospitalization, and likelihood of fatality. In the patients with large burns, increased capillary permeability occurs not only at the injured sites, but also in remote organs. Just as the edema in the burn wound, the respiratory mucosa would develop into edema caused by an inflammatory cascade [7]. The exudation of plasma proteins from the pulmonary microvasculature into the interstitial and alveolar spaces leads to secretion formation which congeal with neutrophils and necrotic cellular debris to form fibrin casts, partially or fully obstructing the airways and causing bronchoconstriction. Induced by the acute inflammatory response, release of the proteolytic enzymes and oxidants destroys the lung parenchyma and increases the microvascular permeability, further accelerating edema formation and decreasing gas exchange [8]. Alveloar collapse and decrease in lung compliance result from diminished surfactant.

In conclusion, burn patients with inhalation injury develop pulmonary complications frequently, including reactive airway disease, acute respiratory distress syndrome (ARDS) and pneumonia. VAP is a common complication of ARDS: about 34 to 70 % of patients with ARDS develop VAP [9].Autopsy demonstrated that nearly 73 % of patients with diagnosis of ARDS also have histological evidence of pneumonia [10].

#### Else predisposing factors

Host factors includes burn/trauma, severe critical illness, age>60 years, ARDS, chronic obstructive pulmonary disease, serum albumin<2.2g/dL, multiple organ dysfunction, large-volume gastric aspiration, gastric colonization, elevated pH, Upper respiratory tract colonization and sinusitis. Intervention factors includes mechanical ventilation>2 d, re-intubation, supine head position, paralytic agents, continuous intravenous sedation, positive end-expiratory pressure, frequent ventilator circuit changes, >4 units of blood products transfused, histamine 2-blockers, antacids, nasogastric tube, prior antibiotic therapy, transport out of the intensice care unit [11, 12].

### Prevention strategies of VAP

#### Necessity of mechanical ventilation

In General, unnecessary intubation and mechanical ventilation should be avoided as much as possible, because this process can increase the risk of VAP 6 to 21-fold. However, when the necessary intubation is needed, it should be performed under controlled conditions as much as possible. The research shows that intubations in emergency department lead to higher incidence of VAP than in the ICU setting [13]. Furthermore, VAP incidence can be reduced when accidental tube dislodgement and emergency reintubation are avoided. In order to limit reintubation for laryngeal edema and postextubation stridor, presence of a cuff leak over 10 to 15 % has been suggested as a simple screnning test whether reintubation is needed or not, and that is particularly useful in the burn patients with extensivr head and neck edema following burn resuscitation. The recent study has showed that daily interruption of sedation and weaning protocols can not only reduce the duration of mechanical ventilation in ICU patients, but also improve the extubation rates without an increase in reintubation rates [14].

#### Specialized endotracheal tubes

Because of reducing the bacterial inoculum, specialized endotracheal tubes may prove particularly beneficial in patients with prolonged mechanical ventilation. In several studies, tubes with continuous aspiration of subglottic secretions have reduced the incidence of early-onset VAP [15]. Because of the broad-spectrum antimicrobial activity of silver, silver impregnated endotracheal tubes have potential applications in theory. Silver-coated endotracheal tubes were used in a randomized trial of 9417 patients across 54 centers in North America. The results show that a 35.9 % relative risk reduction in VAP which reduce the incidence of VAP from 7,5 % to 4.8 % in a mixed ICU population of patients expected to require intubation for over 24 hours [16].

#### Reducing aspiration from gastrointestinal sources

As an increased risk of aspiration of gastric contents, the administration of enteral nutrition with supine position are thougt to be a risk factor for the development of VAP [17]. The research has demonstrated that patients administrated with enteral nutrition in the supine position have as much as a 50 % incidence of VAP compared to 5 % in those kept in the semirecumbent position [18]. Therefore, burn patients with maintaining mechanical ventilation should be kept in the semirecumbent position instead of the supine position especially when administrated with enteral nutrition.

#### Use of prophylactic antibiotic

In several studies, modulation of oropharyngeal colonization has also been shown to reduce the incidence of VAP by using combinations of oral antibiotics, with or without systemic therapy, or selective decontamination of the gastrointestinal tract (SDD) [19]. In the areas where the incidence of VAP is considerablely high, the idea of prophylactic antibiotics to decrease the incidence is similar to SDD. Trimethoprim-sulfamethoxazole (TMP-SMX) could obviously reduce the incidence of VAP in large burn patients which is demonstrated by several researches [20]. However, due to the high endemic levels of antibiotic resistance, the benefits of prophylactic antibiotics for VAP have been considerablely low in ICUs. In this circumstance, prophylactic antibiotics seemed to increase the selective pressure for antibiotic-resistant microorganisms resulting in strong resistance of pathogenic bacteria. Thus, routine prophylactic antibiotics should not be recommended in hospital where there are high levels of antibiotic resistance, although the use of prophylactic antibiotics can reduce early-onset VAP.

#### Reducing oropharyngeal bacterial colonization

Chlorhexidine oral rinse is a simple and cost-effective prevention strategy for VAP in many different patient population which has been shown in many studies. A 58 % reduction in incidences of pneumonia has been found by Houston et al. [21] in patients intubated>24 h with chlorhexidine oral rinse. In critically ill patients ventilated>48 h, there is also a 65 % reduction in VAP by the same way [22]. Recently, the use of selective oropharyngeal decontamination (SOD) has been advocated. However, a recent meta-analysis demonstrated that SOD significantly reduces the incidence of lower respiratory tract infections but does not reduce mortality [23].

#### Restrictive blood transfusion

As we all know, postinjury immunosuppression is considered as a complication of severe burn injury. Common therapies can add to the immune compromised state, such as blood transfusion. Transfusion, which is demonstrated by several studies, can increased the risk for VAP [24, 25]. The American Burn Association has found a 13 % increased risk of infection with each unit of blood transfused in patients with burn injuries>20 % TBSA and 42 % of those transfused developed into VAP [25]. Thus, the restrictive blood transfusion policy should be applied to burn patients in order to reduce infections.

#### Intensive insulin therapy

Controlling blood glucose levels (to levels 4.4-6.1mmol/L) by intensive insulin therapy can decrease mortality in surgical ICU patients which is pulished as a landmark trial in 2001 [26]. However, the subsequent trials did not confirm this benefit. Recently, Hemmila et al. share their experiences of intensive insulin therapy in burn patients. Their adoption of protocol (to levels<7.8mmol/L) did decrease VAP rate with no impact on mortality [27]. The potential benefit of intensive insulin therapy in burn patients needs to be confirmed in more large trials. Before that, the protocol of intensive insulin therapy is an option in burn patients with ventilation.

#### Control environmental infection

The environment, healthcare devices, and even staff can all serve as sources of infection. Bacterial colonization of the ventilator circuit may also lead to VAP. By institution of an infection control protocol , the U.S. military has decreased the incidence of VAP in Irap recently, which includs hand hygiene, chlorhexidine oral care, contact barrier precautions, patient and staff cohorting, and reducing the duration and spectrum of surgical antimicrobial prophylaxis [28].

### Diagnosis of VAP

As to the diagnosis, there is no clinical gold standard existing for the diagnosis of VAP. Many patients with the clinical diagnosis of pneumonia may have no infectious etiologies, while as many as 66 % of patients with the clinical diagnosis of VAP did not meet the microbiologic criteria for infection. In order to improve the ability to diagnosis VAP, the scoring systems such as the U.S. centers for disease control and prevention (CDC) Criteria and the Clinical Pulmonary Infection Score (CPIS) have been established, which combine clinical, radiographic, physiologic (PaO2/FiO2), and microbiologic data, to improve the specificity of VAP diagnosis. An initial score>6 and a persistently elevated CPIS>6 after 3 days of empiric treatment are strongly related to VAP in mediacal and ICU patients. However, the CPIS scale is not able to differentiate inflammation from infection, a differentiation that can be definitively made by quantitative culture (Table 1).

**Table 1:** The clinical pulmonary infection score (CPIS) [36]

Componet	Value	Points
Temperature C	≥36.5 and ≤38.4	0
	≥38.5 and ≤38.9	1
	≥39.0 and ≤36.0	2
Blood leukocytes per mm^3^	≥4000 and ≤11,000	0
	<4000 or >11,000	1
Tracheal secretions	Few	0
	Moderate	1
	Large	2
	Purulent	+1
Oxygenation PaO2/FiO2	>240 or presence of ARDS	0
	≤240 and absence of ARDS	2
Chest radiograph	No infiltrate	0
	Pathy or diffuse infiltrate	1
	Localized infiltrate	2

In contrast to the CPIS, it is a recommended strategy to obtain a lower airway quantitative culture when VAP is suspected, using broncheoalvolar lavage (BAL), protected specimen brush, or a nonbronchoscopic technique (so-called mini BAL). The quantitative strategy can increase specificity of diagnosis and identify the responsible organisms, and reduce the extended use of broad-spectrum antimicrobials in the ICU. Most authors use 1*10 5CFU/ml as BAL threshold, however, several studies in the trauma population have used a BAL threshold of 1*10 5 CFU/ml to limit unnessary administration of systemic antibiotics and rates of VAP recurrence have no significantly differences. Besides, BAL should not be considered as 100 % accurate in identifying the infective bacterium, but could show good correlation [29].

### Treatment of VAP

#### Early Use of broad-spectrum antibiotic coverage

Many studies have shown that a delay in the initiation of appropriate antibiotic therapy for patients with VAP has association with increased morbidity, cost of care, and mortality whether VAP is early or late in onset [30, 31]. Therefore, once a patient is diagnosed with VAP, broad-spectrum antibiotic therapy should be adopted immediately targeting specific likely pathogens based on timing of VAP onset and modified by knowledge of local patterns of antibiotic resistance. Importantly, a quantitative culture should be obtained before adoption of antibiotics, and diagnostic threshold should be lowed if the culture is obtained after administration of antibiotics.

#### De-escalation of broad-spectrum antibiotic

If quantiative culture was obtained, antimicrobial spectrum should be narrowed according to microbiologic results and sensitivities, known as de-escalation. Use of ICU specific, broad-spectrum, empiric therapy is able to lead to a significant increase in the administration of appropriate antimicrobial treatment, a decrease in development of secondary episodes of antibiotic-resistant VAP, and a significant reduction in the duration of antibiotic treatment [32]. Furthermore, a recent international consensus conference agreed that the use of broad-spectrum antibiotics for <48h would not induce a significant risk of multiple drug resistance (MDR).Therefore, de-escalation of broad-spectrum antibiotic should be adopted as soon as quantitative culture results are known [33].

#### Antibiotic rotation

Antibiotic rotation has been evaluated and adopted by many doctors, although no study has specifically examined its role in burns. Several evaluation researches have showed a decreased incidence of MDR bacteria and mortality benefit with the adoption of antibiotic rotation [32]. However, antibiotic rotation should be considered as an option for burn centers to reduce the spread of MDR bacteria until further burn unit specific data become available.

#### Duration of antibiotic treatment

It is suggested by recent data from many other trails that antibiotics can be stopped once clinical signs of infection have resolved. This may also decrease the incidence of secondary pneumonias with MDR organisms. Dennesen et al. have reported that most of the clinical signs of pneumonia have resolved by 6 days of therapy. Another randomized trial in France demonstrated that there is no difference in mortality or recurrent infection in patients treated with an 8 day course of antibiotics compared with those treated with a 15 day course [34]. Additonally, those treated with 8 days of antibiotics had a lower incidence of multiresistant pathogens if a recurrent infection developed. Notebly, if the original infecting organism was a nonlactose fermenting Gram-negative bacilli such as Acinetobacter baumanii, those treated with an 8 day course had a higher pulmonary infection recurrence rate [35]. Similar to the strong virulence of nonlactose fermenting Gram-negative bacilli, MRSA pneumonias are often quite virulent, with demands for longer ICU and ventilator support. Thus, unless caused by MRSA or nonlactose fermenting Gram-nagative rods, in which case the treatment of 15 day course is recommended, antibiotic treatment for VAP should be limited with 8 days.

## Conclusion

Although the research on VAP has made obvious progress in recent years, there is still no clinical gold standard existing for the diagnosis of VAP that requires further researched. The recommendations in these guidelines are derived from available general critical care and burn literature. Although most of the data were obtained by study of medical, surgical, and trauma patients these finding are also applicable to critically ill burn patients with mechanical ventilation.
